# Regulation of the microtubular cytoskeleton by Polycystin-1 favors focal adhesions turnover to modulate cell adhesion and migration

**DOI:** 10.1186/s12860-015-0059-3

**Published:** 2015-05-07

**Authors:** Maddalena Castelli, Chiara De Pascalis, Gianfranco Distefano, Nadia Ducano, Amanda Oldani, Letizia Lanzetti, Alessandra Boletta

**Affiliations:** Division of Genetics and Cell Biology, San Raffaele Scientific Institute, Milan, Italy; Current Address: International PhD Program, Institut Pasteur, Paris, France; Candiolo Cancer Institute, Candiolo, Torino Italy; Department of Oncology, University of Torino, Torino, Italy; IFOM, Fondazione Istituto FIRC di Oncologia Molecolare, Milan, Italy

**Keywords:** Polycystin-1, Migration, Microtubules, Adhesion, Focal adhesion turnover, Focal adhesion kinase

## Abstract

**Background:**

Polycystin-1 (PC-1) is a large plasma membrane receptor, encoded by the *PKD1* gene, which is mutated in most cases of Autosomal Dominant Polycystic Kidney Disease (ADPKD). The disease is characterized by renal cysts. The precise function of PC-1 remains elusive, although several studies suggest that it can regulate the cellular shape in response to external stimuli. We and others reported that PC-1 regulates the actin cytoskeleton and cell migration.

**Results:**

Here we show that cells over-expressing PC-1 display enhanced adhesion rates to the substrate, while cells lacking PC-1 have a reduced capability to adhere. In search for the mechanism responsible for this new property of PC-1 we found that this receptor is able to regulate the stability of the microtubules, in addition to its capability to regulate the actin cytoskeleton. The two cytoskeletal components are acting in a coordinated fashion. Notably, we uncovered that PC-1 regulation of the microtubule cytoskeleton impacts on the turnover rates of focal adhesions in migrating cells and we link all these properties to the capability of PC-1 to regulate the activation state of Focal Adhesion Kinase (FAK).

**Conclusions:**

In this study we show several new features of the PC-1 receptor in modulating microtubules and adhesion dynamics, which are essential for its capability to regulate migration.

**Electronic supplementary material:**

The online version of this article (doi:10.1186/s12860-015-0059-3) contains supplementary material, which is available to authorized users.

## Background

Autosomal Dominant Polycystic Kidney Disease is a common monogenic disorder characterized by the formation of epithelial cysts in the kidney, liver and pancreas. The disease is due to mutations in two genes: the *PKD1* and the *PKD2* genes, mutated in 85 and 15% of cases respectively, which encode for Polycystin-1 (PC-1) and Polycystin-2 (PC-2). PC-1 is a large protein composed by a relatively short intracellular C-terminus (198aa), 11 trans-membrane domains that ensure its localization at ER and cytoplasmic membrane, and a long extracellular N-terminus (≈3000aa) [1]. The C-terminal tail likely mediates a series of signaling pathways [2,3], while the large N-terminal region contains a number of domains possibly involved in mediating protein-protein interaction and/or in sensing mechanical stimuli [4,5]. The protein localizes at cell-cell and cell-matrix contacts as well as at the primary cilium [1]; here, PC-1 is proposed to directly sense urine flow [6] and possibly mediate activation of its partner PC-2, which is a calcium channel of the TRPP family, although this model has been recently challenged [7]. Consistently with the localization at cell-cell junctions it has been shown that Polycystin-1 is involved in cell-cell adhesion dynamics [8,9]. Finally, at the cell-matrix interface PC-1 has been proposed to play a role in cell-substrate adhesion [10] and the short intracellular C-tail of PC-1 has been previously localized into Focal Adhesions (FA) [2]. However, the ability of PC-1 to mediate and control cell adhesion to the substrate has never been investigated in detail, although its role in this context has been one of the first functions proposed for this receptor, and suggested to play a role in ADPKD phenotype [10,11].

The capability of cells to adhere to the substrate is fundamental for many cell biological processes, including key aspects during embryonic development. Cell adhesion to extracellular matrix is a highly dynamic and tightly regulated process [12]. At the front edge of a migrating cell the formation and maturation of multi-protein focal adhesions provide the basis for setting the tension to propel the cell forward. At the cell rear, instead, the disassembly of the FAs mediated by a microtubule-guided process allows free cell movement. Each of these steps is regulated by several proteins, although details of the mechanisms remain elusive. Among all, focal adhesion kinase (FAK) is an important player in these processes [13]: FAK−/− fibroblasts display defective cell migration and an accumulation of immature focal contacts [14,15]. Indeed, FAK directly interacts with adhesion components such as integrins, and phosphorylates paxillin, a fundamental component of focal complexes [16]; overexpression of a mutated form of paxillin which cannot be phosphorylated by FAK prevents the turnover of focal contacts and cell motility [17].

Interestingly, several studies in the past from our and other groups have implicated a role for PC-1 in regulation of different aspects of the migratory process [2,9,18-21]. Indeed Polycystin-1 induces actin cytoskeleton rearrangements and protrusion at the cell edge in wound healing assays, and it also favors the dynamic of cell-cell adhesion, promoting β-catenin turnover [9] in epithelial cells. A dual role for Polycystin-1 in regulation of cell migration has been proposed: PC-1 is able to regulate both the rate of cell movement as well as the orientation of cells during migration [9,18].

Here we report a series of novel observations on the role of PC-1 in cell migration. We report that PC-1 is able to regulate the microtubule stability and dynamics in addition to the actin cytoskeleton. Furthermore, we report that the capability of PC-1 to influence the microtubule cytoskeleton results in a dynamic regulation of focal adhesion formation and in an enhanced adhesion to the substrate. Interestingly, we show that all these effects of PC-1 depend on the activity of FAK and are important for regulation of cell migratory rates. Of interest, we show that the actin cytoskeleton is not essential in PC-1 mediated cell orientation during migration, a process in which the dynamic regulation of the microtubule cytoskeleton is instead essential.

## Results

### Polycystin-1 regulates microtubule stability

**Figure 1:**
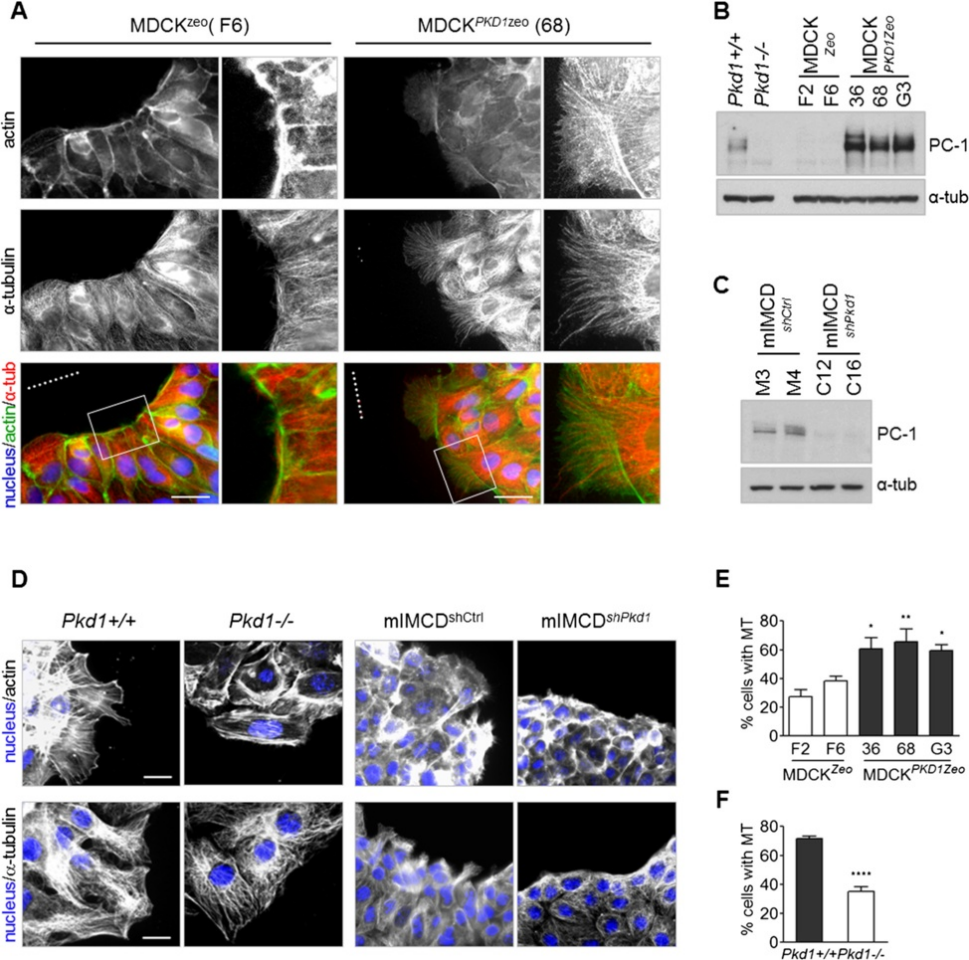
Polycystin-1 levels correlate with cytoskeleton protrusions formation in wound-healing assay and microtubules stabilization. **(A)** MDCK^Zeo^ (clone F6) and MDCK^*PKD1*Zeo^ (clone 68) cells were subjected to wound healing, allowed to migrate for one hour and stained with α-tubulin antibodies (white/red), FITC-phalloidin (white/green) and DAPI staining (blue). Full images and zoom-in of the boxed areas show that MDCK^*PKD1*Zeo^ cells have both actin filaments and microtubules elongated towards the wound. Bar: 20 μm. **(B)** Western blot analysis of PC-1 levels using LRR antibody in fibroblasts *Pkd1* ^*+/+*^ and *Pkd1* ^*−/−*^, and in control MDCK^Zeo^ (clone F2, F6) and PKD1-overexpressing MDCK^*PKD1*Zeo^ (clone 36, 68, G3) epithelial cells. **(C)** Western blot analysis using PC-1 LRR antibody of mIMCD cells: two clones isolated from shScrambled-infected (M3 and M4) and two *shPkd1*-infected (C12 and C16). **(D)** *Pkd1* ^*+/+*^ and *Pkd1* ^*−/−*^ fibroblasts, as well as mIMCD^*shCtrl*^ and mIMCD^*shPkd1*^, were subjected to wound, allowed to migrate for one hour and stained with DAPI (blue) and FITC-phalloidin (white) or α-tubulin antibodies (white). Images show defective protrusions in the two cytoskeletons in *Pkd1* ^*−/−*^ and mIMCD^*shPkd1*^ cells, compared to their respective controls. Bar: 40 μm. **(E)** Quantification of nocodazole resistance assay in MDCK cells, revealing that MDCK^*PKD1*Zeo^ cells have more persistent microtubules compared to MDCK^Zeo^ cells. The graph is representative of three independent experiments; averages and SD are shown. Statistical analysis: ANOVA; *p < 0.05, **p < 0.01, relative to both control clones (F2 and F6). **(F)** Quantification of nocodazole resistance assay in fibroblasts, revealing that *Pkd1* ^*−/−*^ cells have less persistent microtubules compared to *Pkd1* ^*+/+*^ cells. The graph is representative of three independent experiments; averages and SD are shown. Statistical analysis: T-test; ****p < 0.0001.

We have previously reported that Polycystin-1 regulates both the rates of cell migration and front-rear polarity of migrating cells in MDCK epithelial cells and in fibroblasts [9,18]. Actin and microtubule cytoskeletons are directly involved in extending new protrusions in the direction of migration and in generating the propelling force for cell movement. PC-1 overexpression has been reported to induce actin protrusion [9,18]. Therefore, we carefully analyzed actin filaments and microtubules by immunofluorescence staining using phalloidin and an anti-α-tubulin antibody in cellular models of gain or loss-of-function of PC-1 [9,18]. We analyzed cells in wound-healing assays, in which a migration stimulus is generated by scratching the cell monolayer with a pipette tip. 1 hour after wounding, when the migration involves mainly the first row of cells facing the wound, a set of previously described MDCK cells carrying over-expression of PC-1 (MDCK^*PKD1Zeo*^) [22] reveals the presence of actin protrusions and elongated microtubules, while control MDCK cells (MDCK^*Zeo*^) have a strong actin staining at the cell-cell junctions and randomly oriented microtubules (Figure 1A and B). Conversely, fibroblasts carrying null alleles of the *Pkd1* gene (*Pkd1*^*−/−*^) [18] display defective actin and microtubule organizations compared to *Pkd1*^*+/+*^ control cells (Figure 1B and D). To test if the absence of PC-1 is linked with defective actin and microtubular protrusions in the setting of epithelial cells, we generated a set of mIMCD cells carrying stable silencing of the *Pkd1* gene, generating mIMCD^*shPkd1*^ clones carrying a 60 to 70% downregulation of PC-1 (C12 and C16, Figure 1C and Additional file 1) as compared to parental mIMCD cells or controls infected with a scrambled shRNA (mIMCD^*shCtrl*^, clones M3 and M4) (Figure 1C and Additional file 1). Wound healing assays followed by immunofluorescence revealed that mIMCD^*shCtrl*^ cells tend to have more actin and microtubule-based protrusions at the leading edge as compared to mIMCD^*shPkd1*^ cells (Figure 1D).

Since microtubule elongation in oriented cell migration correlates with the presence of persistent microtubules, we hypothesized that PC-1 can influence the rate of microtubule stabilization. To formally test this hypothesis we performed nocodazole resistance assays, which consist in treating living cells with nocodazole (2 μM) for a short period of time, to depolymerize only the newly formed microtubules, followed by fixation and tubulin immunostaining. Quantification revealed that MDCK^*PKD1Zeo*^ cells have increased resistance to nocodazole, when compared to control clones (Figure 1E). In line with this, we found that *Pkd1*^*−/−*^ fibroblasts, show decreased stabilization of the cytoskeleton, as compared to wild-type controls (Figure 1F). We conclude from these studies that PC-1 regulates the microtubules in addition to the microfilaments.

### Both the microtubule and the actin cytoskeletons are involved in *PKD1*-dependent cell migration

**Figure 2:**
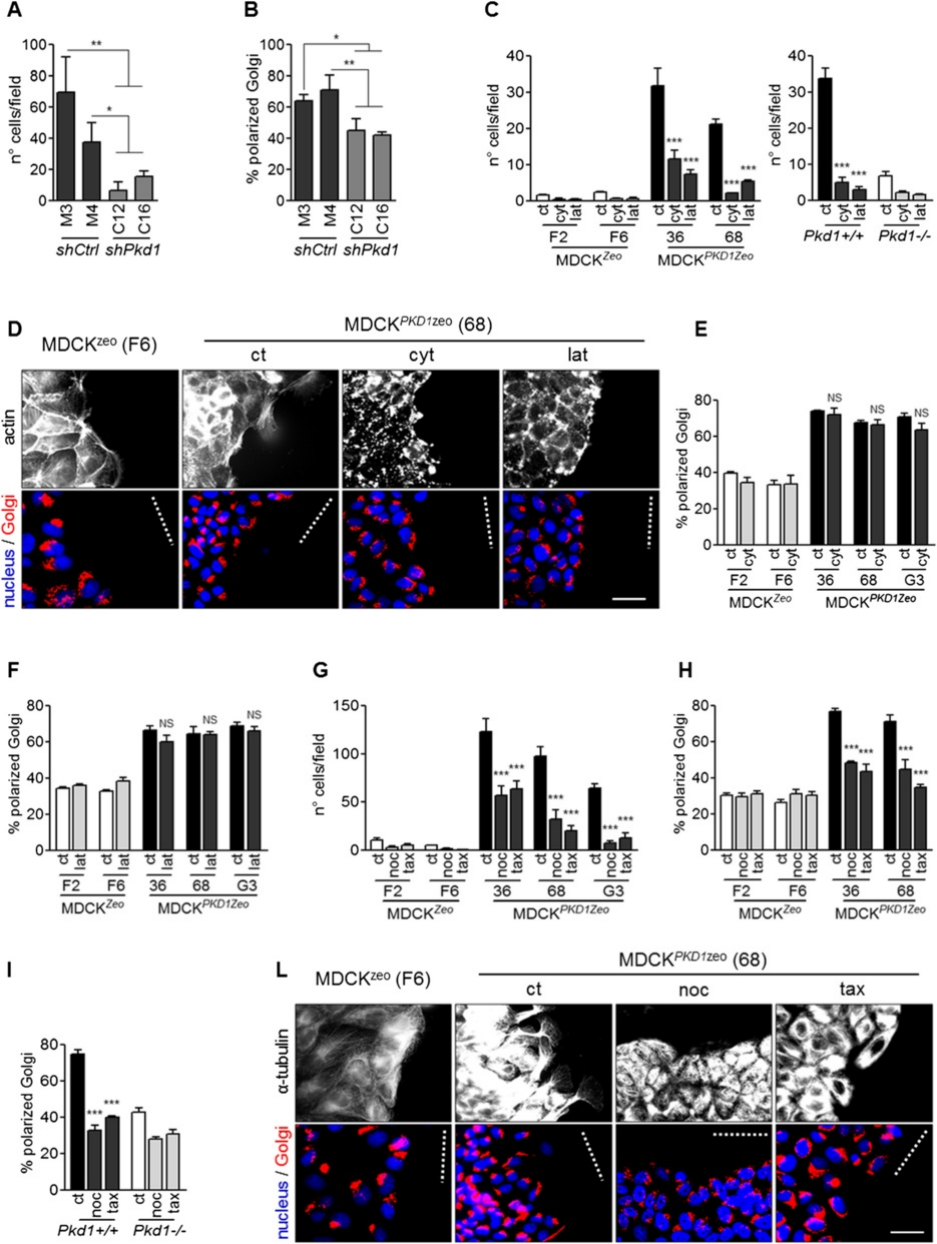
Polycystin-1-dependent migration depends on both actin and microtubules, while front-rear polarity only on microtubules. **(A)** Boyden chambers on mIMCD cells, showed that silencing of *Pkd1* (mIMCD^*shPkd1*^: clones C12, C16) decreases their migration capability, as compared to controls (mIMCD^*shCtrl*^: clones M3, M4). **(B)** Golgi orientation in wound-healing assays on mIMCD cells, showed front-rear polarity impairment upon *Pkd1* silencing. **(C)** Boyden chambers assay performed on MDCK cells (left panel) and fibroblasts (right panel) in the presence of CytochalasinD (cyt) or LatrunculinA (lat), revealed the essential role of actin in migration. Graphs are representative of three independent experiments; averages and SD are shown. **(D)** MDCK^*Zeo*^ (clone F6) and MDCK^*PKD1Zeo*^ (clone 68) were wounded in the presence of CytochalasinD (cyt) or LatrunculinA (lat) and after 3 h stained with phalloidin (actin), anti-giantin (Golgi) and DAPI (nucleus). Bar: 50 μm. **(E-F)** Quantification of Golgi repositioning revealed no effect of CytochalasinD (E, cyt) or LatrunculinA (F, lat) on front-rear polarity in MDCK^*PKD1*Zeo^ cells (clones 36, 68, G3). Graphs are representative of three independent experiments; averages and SD are shown. **(G)** Boyden chamber assays performed in the presence of Nocodazole (noc) or Taxol (tax) revealed the role of microtubules in migration. Graph is representative of three independent experiments; averages and SD are shown. **(H-I)** Quantification of Golgi repositioning revealed a strong effect of Nocodazole and Taxol on front-rear polarity in MDCK^*PKD1*Zeo^ epithelial cells (H) and in *Pkd1* ^*+/+*^ (I). Graphs are representative of three independent experiments; averages and SD are shown. **(L)** Front-rear polarity was evaluated as in **D** in MDCK^*Zeo*^ (clone F6) and MDCK^*PKD1Zeo*^ (clone 68) cells treated with Nocodazole and Taxol. Bar: 50 μm. For all graphs in this figure: Statistical analysis ANOVA; NS non-statistically significant (p > 0.05), *p < 0.05, **p < 0.01, ***p < 0.001, referred to the relative control (ct).

We next aimed at dissecting the relative role of the two cytoskeleton components (microtubules and microfilaments) in PC-1-mediated cell migration. To this end, we used two different assays: Boyden chambers assay to assess cell motility and evaluation of Golgi re-orientation in wound-healing assay to quantify cell orientation during migration. Importantly, in line with our published data, the newly generated mIMCD^*shPkd1*^ cells display both impairment of cell migration in Boyden chambers (Figure 2A) and in front-rear polarity (Figure 2B). Treatment in the presence of CytochalasinD (5 μM) and LatrunculinA (5 μM), both able to interfere with the actin cytoskeleton, hindered *PKD1*-dependent cell migration in Boyden chamber assays in MDCK^*PKD1Zeo*^ cells and in *Pkd1*^*+/+*^ fibroblasts, reducing their motility capabilities down to the levels of MDCK^*Zeo*^ controls and *Pkd1*^*−/−*^ fibroblasts (Figure 2C). On the contrary, neither of the two toxins had effects on the establishment of front-rear polarity (Figure 2D, E and F). Staining of the actin cytoskeleton revealed that both drugs are however able to disrupt the actin filaments, excluding the possibility that the drugs did not effectively act on the actin cytoskeleton (Figure 2D). In previous studies we have shown that the rearrangements in the actin cytoskeleton observed upon over-expression of PC-1 are mediated by the PI-3 kinase cascade. In line with these previous data, we found that the PI-3 kinase inhibitors LY294002 (20 μM) and wortmannin (250nM) are able to inhibit cell migration in Boyden chamber assays, but had no effect on front-rear polarity establishment (Additional file 1), similarly to the actin depolymerizing drugs.

Next, we used nocodazole which is able to interfere with the nascent microtubules when used at nanomolar concentrations (150nM), as well as taxol (1 μM), which instead prevents disassembly of the already polymerized tubulin filaments. Both these drugs were able to strongly interfere with the capability of cells to move across the membrane in Boyden chamber assays (Figure 2G) and to achieve front-rear polarity (Figure 2H, I and L). We conclude that PC-1 regulates both the actin and the microtubules cytoskeletons, but while PC-1 mediated cell motility depends on both the actin and the microtubular cytoskeleton, PC-1-induced front-rear polarity exclusively depends on the latter.

### Polycystin-1 regulates cell adhesion and nascent focal adhesions

**Figure 3:**
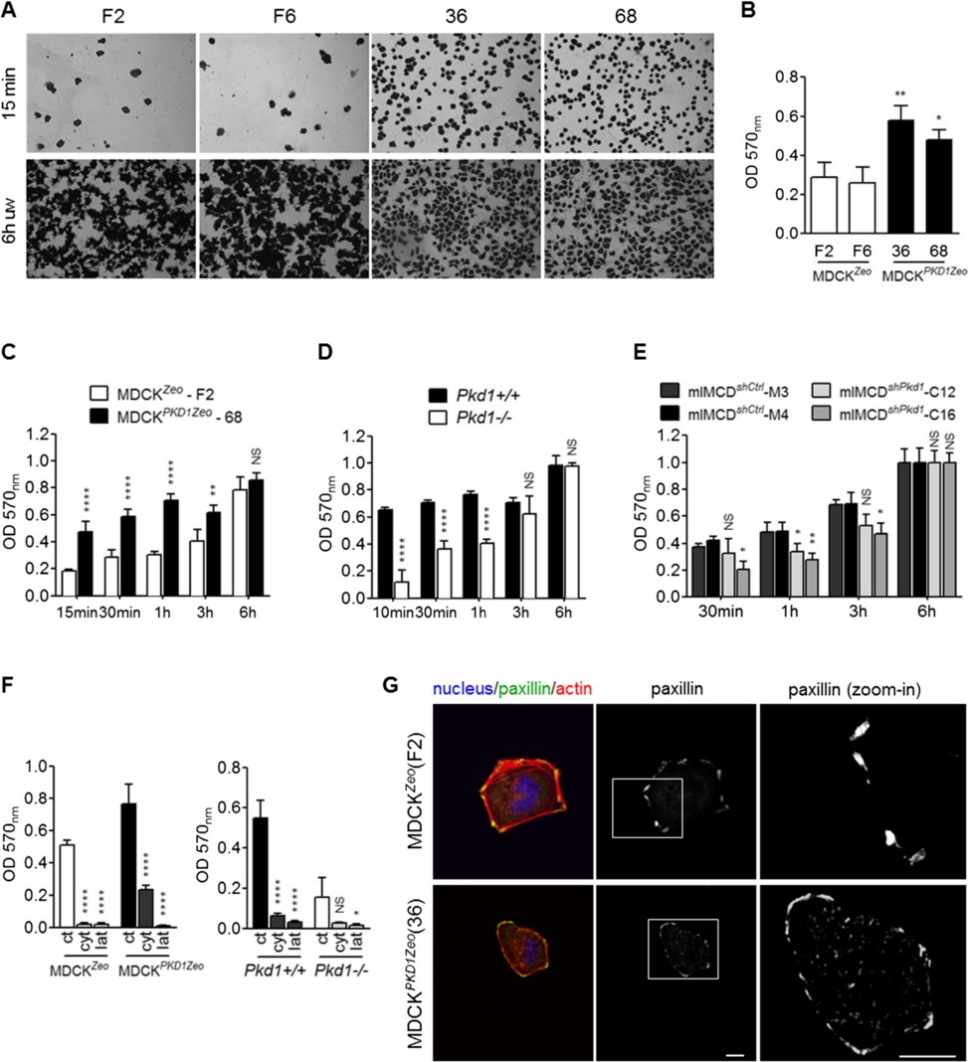
Polycystin-1 regulates cell adhesion to the substrate. **(A)** Images of the colorimetric adhesion assay of MDCK^*Zeo*^ (clones F2, F6) and MDCK^*PKD1Zeo*^ (clones 36, 68) cells upon wash-out. As control, images of unwashed cells are shown. **(B)** Experiments as in **A** are quantified by measuring absorbance (OD 570 nm) of colored cells revealing that MDCK^*PKD1Zeo*^ cells adhere more to fibronectin. The graph is representative of four independent experiments; averages and SD are shown. Statistical analysis: ANOVA; *p < 0.05, **p < 0.01, relative to both control clones (F2, F6). **(C-E)** Time course of a colorimetric adhesion assay with: MDCK^*Zeo*^ (clone F2) and MDCK^*PKD1Zeo*^ (clone 68) cells **(C)**, *Pkd1* ^*+/+*^ and *Pkd1* ^*−/−*^ fibroblasts **(D)**, mIMCD^*shCtrl*^ (clones M3, M4) and mIMCD^*shPkd1*^ (clones C12, C16) cells **(E)**; cells plated on fibronectin were washed at the indicated time points and values normalized for the 6 h unwashed values. Graphs are representative of three independent experiments, performed in triplicate; averages and SD are shown. Statistical analysis: ANOVA; NS non-statistically significant (p > 0.05), *p < 0.05, **p < 0.01, ****p < 0.0001, referred to the control (F2, *Pkd1* ^*+/+*^, mIMCD^*shCtrl*^) relative bars. **(F)** Quantification of the colorimetric adhesion assay in MDCK^*Zeo*^ (clone F2) and MDCK^*PKD1Zeo*^ (clone 68) cells, or in *Pkd1* ^*+/+*^ and *Pkd1* ^*−/−*^ fibroblasts, in presence of cytochalasin (cyt, 5 μM) and latrunculin (lat, 5 μM). revealing an essential role of actin in adhesion. Graph is representative of three independent experiments. Averages and SD are shown. Statistical analysis: ANOVA; NS non-statistically significant (p > 0.05), *p < 0.05, ****p < 0.0001, referred to the relative control (ct). **(G)** Staining of MDCK^Zeo^ (clone F2) and MDCK^*PKD1*Zeo^ (clone 36) cells for actin (phalloidin-TRITC, red), paxillin (green) and nucleus (DAPI, blue) after 1 h on fibronectin. Zoom-in of the boxed areas are shown. Bar: 25 μm.

During the course of our experiments we noticed that MDCK^*PKD1Zeo*^ cells appeared to have a different capability to adhere to the substrate. To formally test if PC-1 can indeed modulate the rate of cell adhesion to the substrate, we performed a colorimetric adhesion assay: briefly, 15 minutes after plating, cells are washed with fresh medium so that all non-adherent cells are removed, while cells that have adhered are fixed, colored and counted. We found that compared to control MDCK^Zeo^ clones, a higher proportion of MDCK^*PKD1Zeo*^ cells still adhere to the plate after the washout (Figure 3A). Colorimetric quantification showed that adhesion is significantly higher in MDCK^*PKD1Zeo*^ clones compared to controls (Figure 3B and C). Consistently with these data, adhesion assays performed on fibroblasts uncovered that *Pkd1*^*−/−*^ and mIMCD^*shPkd1*^ cells have a reduced capability to adhere to the substrate, compared to their respective controls (Figure 3D and E). The difference in adhesiveness is no longer statistically significant only 3 hours after plating in MEFs, while 6 hours after plating in MDCK and mIMCD cells (Figure 3C, D and E), indicating that PC-1 controls the early steps of cell adhesion. Of interest, interfering with the actin cytoskeleton abolished PC-1 effects on adhesion (Figure 3F).

We next asked if the difference in adhesiveness is indeed due to a different capability to assemble focal adhesions. To test this, we analyzed by immunofluorescence cells at early time points after plating (60 minutes), when they are initially spreading on fibronectin matrix. In control MDCK cells, paxillin localizes mainly at the cell periphery and only forms relatively small clusters, very distant from one another. On the contrary, MDCK^*PKD1Zeo*^ cells form many more focal adhesions at the cell periphery, as well as some clusters in the body (Figure 3G). In both cell lines, focal adhesions indeed localize on the basal side of the cell, mediating the contact with the extracellular matrix as expected (Additional file 1).

These data define a role for Polycystin-1 in regulating focal adhesions during the first phases of cell adhesion, explaining the capability to modulate adhesion to the substrate.

### Polycystin-1 regulates focal adhesions during cell migration

**Figure 4:**
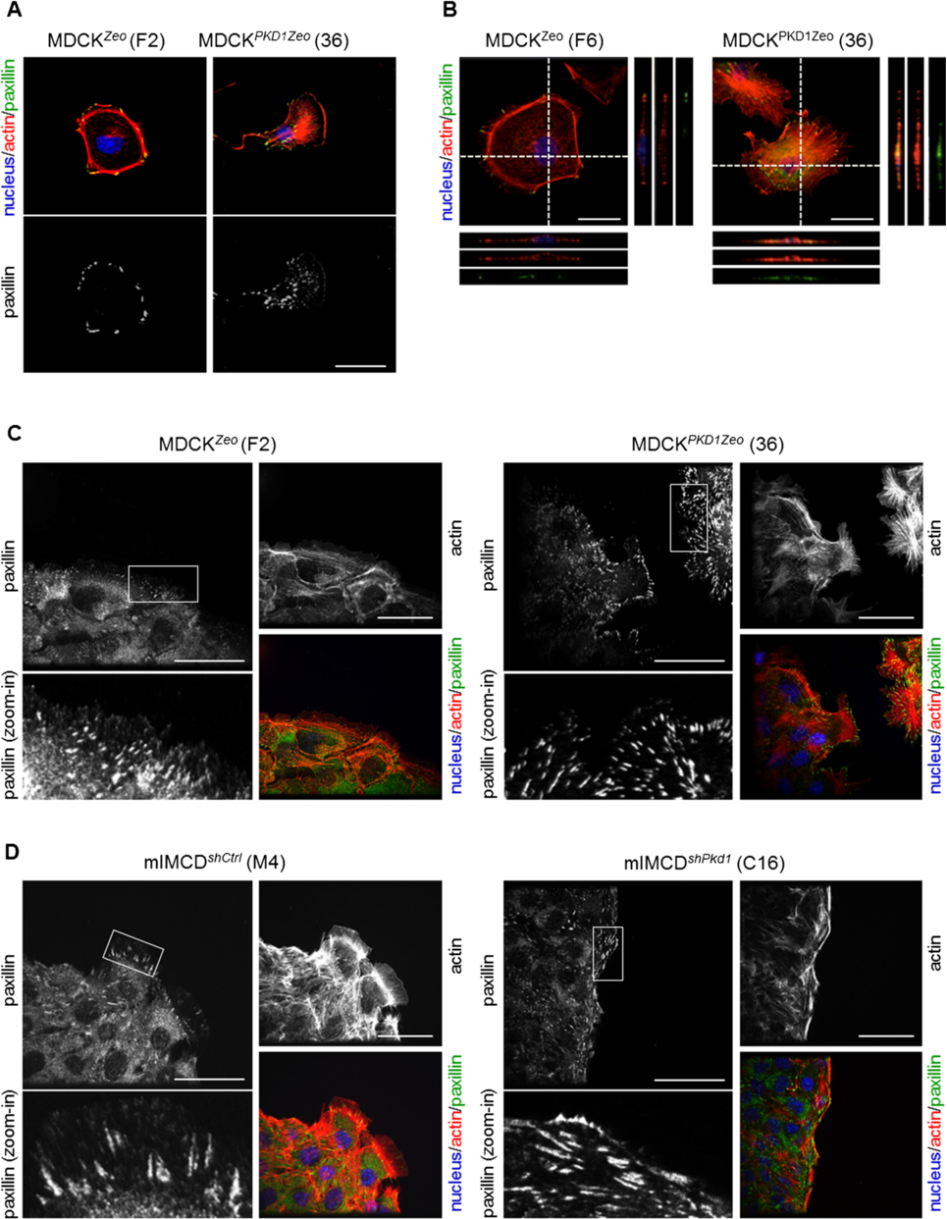
Polycystin-1 modulates focal adhesions distribution during migration. **(A)** Immunofluorescence images of MDCK^Zeo^ (clone F2) and MDCK^*PKD1*Zeo^ (clone 36) cells plated for 5 h on fibronectin. Cells were stained for actin (Phalloidin-TRITC, red), paxillin (green) and nucleus (DAPI, blue). MDCK^Zeo^ cells have a round shape and peripheral clusters of paxillin; MDCK^*PKD1*Zeo^ cells acquire a polarized and migratory phenotype, with focal adhesions of different dimensions. Bar: 25 μm. **(B)** Confocal images of immunofluorescence on MDCK^Zeo^ (clone F6) and MDCK^*PKD1*Zeo^ (clone 36), plated overnight on fibronectin. Cells were stained for actin (Phalloidin-TRITC, red), paxillin (green) and nucleus (DAPI, blue). Images represent one confocal Z-section of the cell, on the right and below each image are projections along x and y axis, reconstructed with Volocity software. In all cell lines, paxillin staining is found on the ventral side and localizes in clusters (focal adhesions). MDCK^*PKD1Zeo*^ cells maintain a polarized morphology and present numerous focal adhesions, while MDCK^*Zeo*^ cells have a round shape and a limited number of paxillin clusters, mainly localized at the periphery of the cell. Bar: 15 μm. **(C-D)** Representative confocal images of MDCK^Zeo^ (clone F2) and MDCK^*PKD1*Zeo^ (clone 36) cells **(C)**, and of mIMCD^*shCtrl*^ (clone M4) and mIMCD^*shPkd1*^ (clone C16) cells **(D)**, allowed to migrate in a 3 hours wound-healing assay on fibronectin and subsequently stained for actin (Phalloidin-TRITC), paxillin (green) and nucleus (DAPI, blue). Merged images, single channels and zoom-in of the boxed areas of paxillin are shown. Bar: 25 μm.

We next investigated the role of PC-1 on focal adhesion distribution during cell migration. First, we performed immunofluorescence analysis of MDCK clones migrating as single cells, 5 hours after plating on fibronectin. Control MDCK^*Zeo*^ cells show a round non-polarized shape with large focal adhesions mostly localized at the peripheral actin bundles (Figure 4A). On the contrary, MDCK^*PKD1Zeo*^ clones acquire a polarized shape and have a higher number of focal adhesions of different sizes: the larger focal adhesions are present in the central-rear part of the cell body, while the smaller ones are localized at the actin protrusions of the front edge (Figure 4A). The same difference in paxillin pattern between control and *PKD1* overexpressing cells is also visible at later time points of migration (Figure 4B). This effect is not correlated with gross clustering differences of focal adhesions, that appear to be correctly formed at the very basal site of the cell in all the clones (Figure 4B). Consistently with these observations in MDCK cells, *Pkd1*^*−/−*^ fibroblasts have predominantly bigger focal adhesions randomly localized, while in wild-type fibroblast paxillin staining reflects the single cell migration pattern (Additional file 1).

Next, we analyzed focal adhesions distribution during collective cell migration, in wound healing assays [9]. After 3 hours from wounding, MDCK control clones present a low number of small focal adhesions, localized near the cell edge but not directly at the tips (Figure 4C and Additional file 1); on the contrary, in MDCK^*PKD1Zeo*^ paxillin staining localizes at the very end of the actin protrusion tips (Figure 4C and Additional file 1). Consistently, focal adhesions at the leading edge of control mIMCD^*shCtrl*^ cells are oriented in the direction of the wound, while *Pkd1* silenced mIMCD^*shPkd1*^ cells show large focal adhesions parallel to the wound (Figure 4D). From these data we conclude that during the process of single cell and collective migration PC-1 is involved in regulating focal adhesion distribution.

### Polycystin-1 regulates focal adhesions turnover

The data above suggest that PC-1 may increase the dynamic of focal adhesion formation.

**Figure 5:**
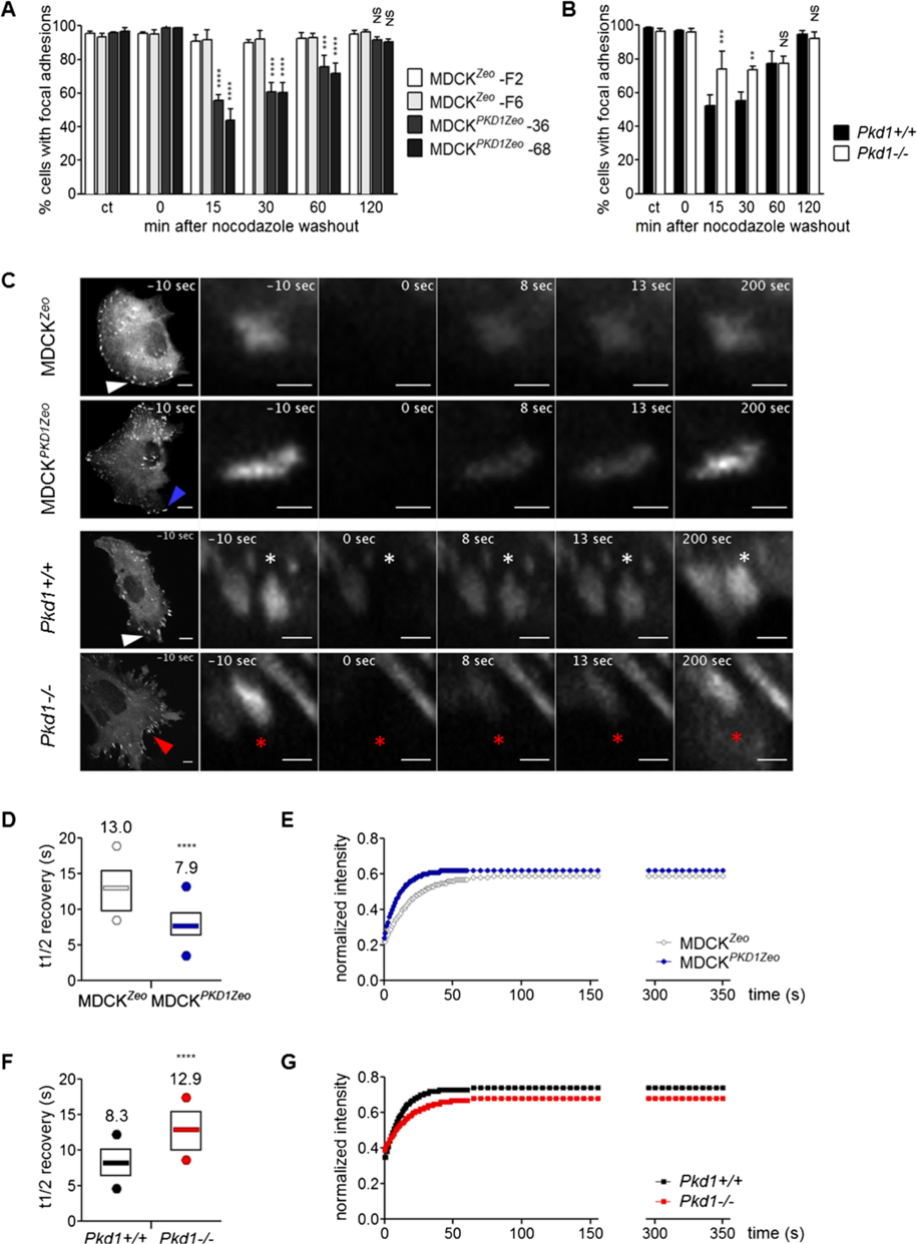
Polycystin-1 regulates focal adhesion dynamics. **(A-B)** Quantification of focal adhesion disassembly in MDCK^Zeo^ (clones F2 and F6) and MDCK^*PKD1*Zeo^ (clones 36 and 68) cells **(A)**, and in *Pkd1* ^*+/+*^ and *Pkd1* ^*−/−*^ fibroblasts **(B)**. Each graph is representative of three independent experiments; in each experiment 3 groups of at least 100 cells were counted. Averages and SD are shown. Statistical analysis: ANOVA; NS (non-significant) p > 0.05, ** p < 0.01,***p < 0.001, ****p < 0.0001, referred to the relative control bars. **(C-G)** MDCK^*Zeo*^ (clone F6) and MDCK^*PKD1Zeo*^ (clone 36), as well as *Pkd1* ^*+/+*^ and *Pkd1* ^*−/−*^ fibroblasts, were transfected with GFP-paxillin. GFP-paxillin-positive adhesions were subjected to FRAP analysis. The panel **(C)** show images of whole cells (bar: 10 μm) and zoom-in of the individual focal adhesions (bar: 2 μm) at crucial time points: pre-bleaching time (−10s), at bleaching completed time (0 s), post-bleaching corresponding at about t1/2 recovery times of the different cell lines (8 s, 13 s), and at long time point (200 s). Bleached focal adhesions are indicated with arrows in the first image and asterisks in the higher magnification images. The results showing the half time (t1/2) of fluorescence recovery are reported in the box plots **(D, F)**; average values are indicated above each box. Statistical analysis: t-test, ****p < 0.0001. F6, n = 22; 36, n = 30, *Pkd1* ^*+/+*^, n = 16; *Pkd1* ^*−/−*^, n = 28 (n, number of focal adhesion analyzed). In the box plot: the line indicates the median value, the box indicates the 1st and 3rd quartile values, the points indicate the minimum and maximum values. Sample fluorescence recovery curves of FRAP are shown in **(E)** and **(G)**. The fluorescence intensity in the recovery curves corresponds to the fluorescence at each time point after photobleaching, background subtracted, and normalized to the pre-bleaching intensity.

To test this hypothesis we first analyzed focal adhesion disassembly with nocodazole washout assays [23]. Briefly, nocodazole (10 μM) is added to the medium of serum-starved cells to completely depolymerize microtubules, and then washed out with fresh serum-starvation medium to allow microtubule regrowth; as microtubules start to target focal adhesion these are disassembled. The staining of paxillin and the related quantification showed that in MDCK^*PKD1Zeo*^ cells the majority of focal adhesions are disassembled 15 minutes after nocodazole washout; on the contrary, controls do not lose focal adhesion structures in these conditions (Figure 5A; Additional file 1). This difference is not secondary to a difference in microtubule depolymerization: indeed, in both clones, microtubules are completely depolymerized by nocodazole treatment and start to reform with the same timing, 15 minutes after nocodazole washout (Additional file 1); focal adhesions are completely reformed 2 hours later (Figure 5A). Next, we looked at the loss-of-function cellular systems. Similar analysis in MEFs revealed that *Pkd1*^*−/−*^ cells maintain their paxillin staining 15 minutes after nocodazole washout, while *Pkd*^*+/+*^ cells have lost their focal adhesion structures (Figure 5B and Additional file 1).

To more precisely assess if PC-1 presence or absence correlates with the rate of Focal Adhesions turnover, we tested the time required for a full turnover of paxillin using Fluorescence Recovery After Photobleaching (FRAP) on a transfected GFP-tagged paxillin molecule [24] (Figure 5C). The mean half-time of fluorescence recovery (FRAP t1/2) in the bleached area was determined as an estimate stability of adhesion binding. MDCK^*PKD1Zeo*^ cells exhibited a significantly faster recovery of GFP-paxillin compared to control MDCK^*Zeo*^ cells (Figure 5C, D and E; Additional files 2 and 3). Consistently, the opposite was observed in *Pkd1*^*−/−*^ fibroblasts as compared to *Pkd1*^*+/+*^ (Figure 5C, F and G; Additional files 4 and 5).

These data demonstrate that Polycystin-1 positively regulates focal adhesion disassembly in the experimental conditions used and suggest that the capability of PC-1 to regulate the microtubule cytoskeleton stability is likely involved in regulation of the turnover of focal adhesions, ultimately mediating cell adhesion and migration.

### Focal adhesion kinase is important for *PKD1*-dependent cell migration and adhesion

**Figure 6:**
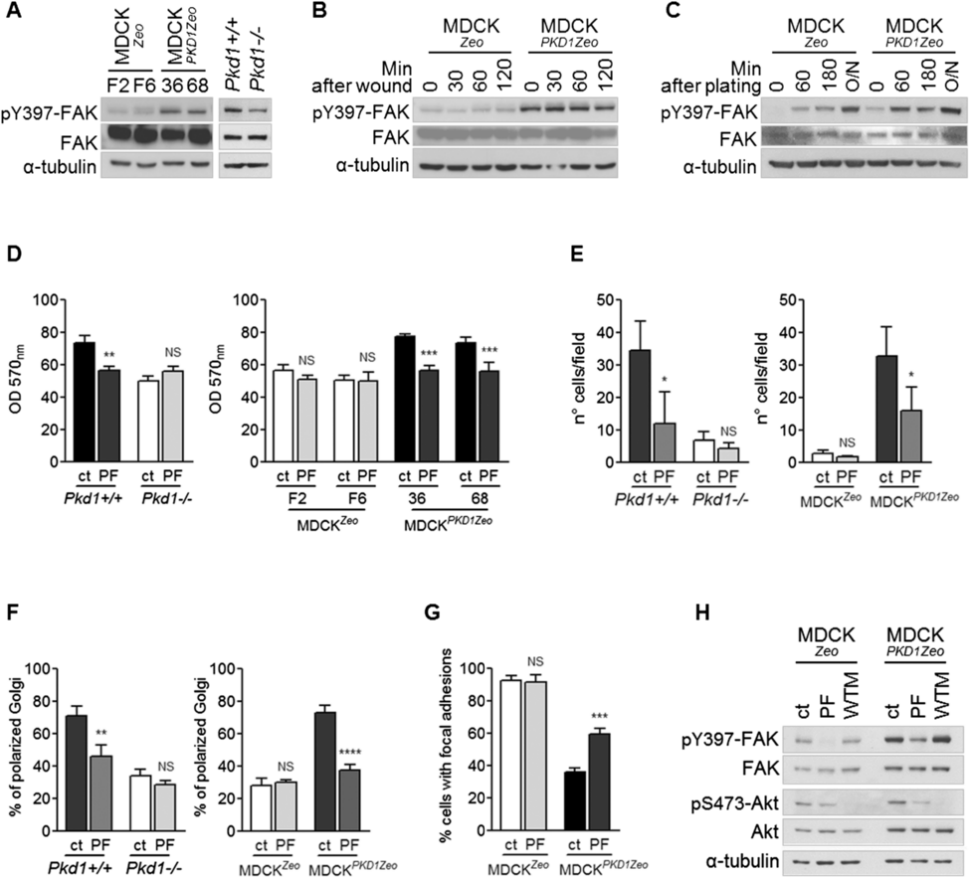
Polycystin-1 induces Focal Adhesion Kinase (FAK) activation, important for cell adhesion, migration and front-rear polarity. **(A)** Western blot analysis in MDCK cells (left panel) and fibroblasts (right panel), after overnight plating at 50% confluence reveals higher phosphorylation, i.e. activation, of FAK in MDCK^*PKD1*Zeo^ cells compared to MDCK^Zeo^, and lower in *Pkd1* ^*−/−*^ compared to *Pkd1* ^*+/+*^ fibroblasts. **(B-C)** Western blot analysis as in **A** of cells collected at different time points after wounding **(B)** or after plating **(C)**. **(D)** Colorimetric adhesion assay performed in the presence of the FAK inhibitor PF-228 (PF, 10 μM) revealed a significant decrease in adhesion of both *Pkd1* ^*+/+*^ and MDCK^*PKD1Zeo*^ cells. Graphs are representative of three independent experiments. Averages and SD are shown. Statistical analysis: ANOVA; NS (non-significant) p > 0.05, **p < 0.01, ***p < 0.001. **(E)** Boyden chamber assays performed in presence of PF-228 (PF, 10 μM) results in a significant decrease in migration of *Pkd1* ^*+/+*^ and MDCK^*PKD1Zeo*^ cells. Averages and SD are shown. Statistical analysis: ANOVA; NS (non-significant) p > 0.05, *p < 0.05. **(F)** Golgi orientation in wound-healing assays in the presence of PF-228 (PF, 10 μM) show a significant decrease in front-rear polarity of *Pkd1* ^*+/+*^ fibroblasts and MDCK^*PKD1Zeo*^ cells. Averages and SD are shown. Statistical analysis: ANOVA; NS (non-significant) p > 0.05, **p < 0.01, ****p < 0.0001. **(G)** PF-228 (PF, 10 μM) significantly decreases focal adhesion disassembly in MDCK^*PKD1*Zeo^ cells evaluated by nocodazole washout experiments. The graph is representative of three independent experiments. Averages and SD are shown. Statistical analysis: ANOVA; NS (non-significant) p > 0.05, ***p < 0.001, referred to the relative control (ct) bar. **(H)** Western blot analysis reveals that PF-228 (PF, 10 μM) inhibits phosphorylation of the PI3kinase target Akt, while Wortmannin (WTM, 2.5 μM) does not affect FAK phosphorylation.

A fundamental effector of focal adhesion signaling is Focal Adhesion Kinase (FAK), whose activity is marked by its phosphorylation in Tyr397 [14,25]. We analyzed the activity of focal adhesion kinase in our cellular models. We first considered single cell migration conditions, and lysed cells plated overnight at low cell density (50%). In line with the effects on focal adhesions described above, western blot analysis showed that MDCK^*PKD1Zeo*^ have higher levels of Tyr397-phosphorylated FAK compared to control cells and that subconfluent *Pkd1*^*−/−*^ cells have lower phosphorylation levels of FAK than *Pkd1*^*+/+*^ (Figure 6A).

We also tested FAK phosphorylation levels in conditions of collective cell migration, analyzing confluent cells in response to wound. Also in these conditions, MDCK^*PKD1Zeo*^ cells have enhanced phospho-FAK levels as compared to controls, which is visible prior to challenging with the wound and is maintained upon wounding at all the time points analyzed (Figure 6B). These results show that in different migration conditions, Polycystin-1 expression correlates with higher activation of FAK.

Next, to assess if differences in FAK phosphorylation can also be observed in the initial phases of cell adhesion, we serum-starved cells and plated them on fibronectin and collected them at different time points. MDCK^*Zeo*^ control cells show a small increase of FAK phosphorylation after plating. However, MDCK^*PKD1Zeo*^ cells display a much higher activation rate of FAK in all the time points considered (Figure 6C). These data are consistent with the increased rates of adhesion of these cells.

To test if FAK activity is necessary for MDCK^*PKD1Zeo*^ cell adhesion, we performed adhesion assays in the presence of a specific kinase inhibitor (PF-228, 10 μM) [26]. The inhibitor decreases adhesion of both MDCK^*PKD1Zeo*^ clones and *Pkd1*^*+/+*^ fibroblasts (Figure 6D), suggesting that early adhesion to fibronectin mediated by PC-1 depends on this kinase. Importantly, PF-228 also had a profound effect on the capability of cells to migrate (Figure 6E) and to achieve front-rear polarity in wound healing assays (Figure 6F). Next, we tested if FAK inhibition also impairs PC-1 dependent increase in focal adhesion disassembly. Indeed, nocodazole washout assays performed in the presence of PF-228 inhibitor revealed that the disassembly of focal adhesion in MDCK^*PKD1Zeo*^ cells is sensitive to this drug (Figure 6G). Interestingly, PI-3 kinase does not seem to mediate the regulation of FAK phosphorylation in these conditions, as the PI-3 kinase inhibitor wortmannin does not revert PC-1 induced upregulation of FAK (Figure 6H).

## Discussion

Previous studies from our and other groups have implicated PC-1 in mediating cell migration and regulation of the actin cytoskeleton [9,18,20].

We have previously shown that PC- 1 is able to regulate two distinct aspects of cell migration: cell polarity (front-rear polarity, i.e. orientation during migration) and cell motility. *Pkd1* mutants display defective Golgi orientation in wound-healing assay and defects in persistent cell migration [18], defining a role for PC-1 in front-rear polarity establishment. Second, overexpression of the full length *PKD1* cDNA or the only C-terminus of PC-1 protein increases cell migration rate in epithelial cells [9,21], implicating PC-1 in cell motility.

In the present study we show that PC-1 not only acts by enhancing actin cytoskeleton protrusions [9], but it also regulates both elongation and stabilization of the microtubules. We also discerned the relative involvement of the two cytoskeletal components in PC-1-dependent cell migration, uncovering that cell motility requires both actin and the microtubules, while front-rear polarity depends only on the dynamics of the microtubule cytoskeleton. Interestingly, a well documented relationship between the actin and the microtubule cystoskeleton has been described in many different cellular processes, including cell migration [27]. During cell migration the actin cytoskeleton pushes the edge of the cell forward and allows for contraction and retraction of the tail. This process intimately depends on the capability of cells to assemble and disassemble focal contacts. The microtubule cytoskeleton plays a key role in this last process by facilitating exocytosis and endocytosis of key adhesion molecules. Thus, it is not surprising that we find in this study that the coordination of the two types of cytoskeletons is crucial for PC-1 induced migration.

Furthermore, we uncover an essential function of PC-1 in modulating the adhesive properties of cells to the substrate. This property is linked to PC-1 ability to regulate the dynamic formation of FAs, although this must be a fine regulation, since cells lacking the *Pkd1* gene do finally form focal adhesions properly. In addition, during the initial phases of cell adhesion, the PC-1-deficient cells display lower focal adhesion number and higher heterogeneity, further suggesting that PC-1 regulates focal adhesion dynamics and localization. The role of cell adhesion dynamics in cell migration is well recognized. Adhesion to the substrate at the front of the cell generates the tension forces required to propel the cell forward, whereas disassembly of focal adhesions at the cell rear allows retraction of the tail [28]. Importantly, these properties must be balanced within the cell since a misbalance towards focal adhesions formation and maturation can prevent cell motility, while an excessive disassembly might decrease the proper intracellular tension. In migrating cells, *PKD1* overexpression increases focal contacts at the cell protrusive edge and the size of focal adhesions in the tail. We further observed that PC-1 deficient cells show defective focal adhesions disassembly, which explain the presence of large focal adhesions in these cells. Indeed, detailed analysis using Fluorescent Recovery after Photobleaching (FRAP) in cells transfected with a GFP-paxillin molecule as previously described [24], evidenced that PC-1 can influence the rate of focal adhesions turnover. In particular, cells carrying over-expression of PC-1 have an enhanced turnover, while cells lacking PC-1 have a reduced turnover.

The role of PC-1 that we observe in favoring focal adhesion turnover is most likely exerted by Focal Adhesion Kinase (FAK) activity. FAK is a master regulator of adhesion; in particular, it appears to be involved mostly in focal adhesion dynamic and disassembly, more than in their formation or maturation [13]. Consistently, FAK activation is higher in *PKD1* overexpressing cells, and is lower in *Pkd1* mutants. Furthermore, its inhibition decreases PC-1-dependent focal adhesion disassembly and is able to revert PC-1 capability to regulate cell migration acting both on the motility and on front-rear polarity. Our data taken together suggest that FAK might act quite upstream in PC-1-mediated cell migration. Moreover, in line with our previously reported data, while inhibitors of PI-3 kinase are able to revert cell motility, but not front-rear polarity, FAK inhibitors act at both levels. In addition the PC-1 mediated upregulation of the PI-3 kinase/Akt signaling pathway that we have previously described [29] is inhibited by FAK inhibitors, while PI-3kinase inhibitors are unable to revert FAK upregulation. These data taken together suggest that FAK acts upstream of PI-3 kinase/Akt in the PC-1 regulated pathway, although further studies will be required to better understand the molecular details of this regulation.

## Conclusions

In this study we have uncovered a previously unrecognized, though hypothesized, role for PC-1 in regulating cell adhesion and the dynamics of focal adhesion contacts via its capability to regulate the microtubule cytoskeleton. Furthermore, we show that PC-1 can modulate the activity of the focal adhesion kinase (FAK) to exert its function on cell adhesion and migration.

One important question that rises from our study is in which physiological context this role of PC-1 might be important? While additional studies will be necessary to answer this question, here we speculate that this might be relevant during renal development. We have recently shown that during development of the kidney, the morphogenesis of renal tubules is achieved by convergent extension-like movements which are impaired in *Pkd1* mutant tubules [18,30,31]. We speculate here that the cellular movements required during this process might require that PC-1 is able to properly regulate the cytoskeletons and the adhesiveness of cells to the substrate for cells to achieve proper intercalation and eventually convergent extension.

## Methods

### Cell culture

Cells (MEF-Mouse Embryonic Fibroblasts [9,18]; MDCK-Madin Darby Canine Kidney [22] and mIMCD3 lines) were grown in 37°C, 5% CO_2_ incubators, in high glucose medium (Gibco, 41965), 10% FBS (Gibco, 10270), 1% Penicillin-Streptomycin (Life technologies, 15070); for MDCK medium was supplied with 0.5 μg/ml Geneticin (Life technologies, 11811) and 0.05 μg/ml Zeocin (Life technologies R25001). For mIMCD clones medium was supplemented with 1 μg/ml puromycin (Invitrogen).

### Generation of murine Inner Medullary Collecting Duct Cells (mIMCD) silenced for *Pkd1*

Pre-screening of shRNAs targeting murine *Pkd1* was performed as follow: 500,000 *Pkd1*^*HA/HA*^ [32] MEFs were seeded onto p100 dish and after 24 h transduced with viral vectors expressing shRNA encoding scrambled (shScr) sequences (MISSION TRC2 Control Transduction Particle puro Non-Target shRNA 3,8x10^6^ TU/ml SHC202V from SIGMA) or 6 different *Pkd1*-targeting shRNA (shPkd1 a-e) sequences (MiSSION Lentiviral Transduction Particles SHCLNV from SIGMA, batch A TRCN0000302260 4,0x10^7^ TU/ml, batch B TRCN0000304611 3,2x10^7^ TU/ml, batch C TRCN0000304612 1,8x10^7^ TU/ml, batch D TRCN0000304664 1,9x10^7^ TU/ml batch E TRCN0000331808 3,1x10^7^ TU/ml) using MOI (Multiplicity of infection) 1. 48 h after transduction cells were collected, lysed and analyzed by SDS-PAGE on a 3-8% gradient gels (Invitrogen) before transfer onto a PVDF membrane (Millipore). Membranes were probed with primary : anti-HA (Roche) and anti-tubulin (Sigma) antibodies. Pre-screening of shRNAs targeting murine *Pkd1*, identified shPkd1C as the most efficient (Additional file 1). For stable transduction, 200,000 murine Inner Medullary Collecting duct (mIMCD) cells were seeded into 6-well plate and grown in DMEM (Invitrogen), supplemented with 10% v/v Fetal Calf Serum and 1:100 Penicillin 5000 U/ml/ 5000 μg/ml Streptomycin solution (Invitrogen). mIMCD cells were transduced with viral vectors expressing shRNA encoding scrambled sequences (shScr) or the selected *Pkd1*-targeting shRNA sequence (shPkd1C) under puromycin selection, using MOI (Multiplicity of infection) 2 or 4 (Additional file 1). 48 h after transduction, cells were splitted and medium containing 1 μg/ml puromycin (Invitrogen) was added. Untransduced cells were treated with the same puromycin concentration to establish the maximal toxicity of puromycin. After 5 days no untransduced cells survived, and selection of puromycin resistant cells was concluded. Resistant cells were analyzed for PC-1 expression levels as above. Resistant cells treated with MOI 4 and diluted 9:10 were chosen for subcloning. For subcloning 1000 cells were seeded into a p100 dish and 18 clones for shPkd1C and 6 clones for shScr were selected and picked. At confluency cells were analyzed for PC-1 expression levels. Two clones carrying high silencing levels (C12 and C16) and two control clones (M3 and M4) were selected for further use (Figure 1C).

### Inhibitors and antibodies

CytochalasinD (C8273), LatrunculinA (L5163), Nocodazole (M1404) and Taxol (T7402) were from Sigma; FAK inhibitor II/PF 573–228 (324878) and Wortmannin (681675) were from Calbiochem; LY-294002 (V120A) was from Promega.

Fluorescein Isothiocyanate-labeled Phalloidin (P5282), Tetramethylrhodamine B isothiocyanate-labeled Phalloidin (P1951), antibodies for α-tubulin (T5168) and acetylated tubulin (T6793) were from Sigma; DAPI (sc-3598), antibodies for PC-1 (sc-130554) and FAK (sc-558) were from Santa Cruz; antibody for phospho Y397-FAK (44624G) was from Life technologies; paxillin (610052) antibody was from BD Bioscience; antibody for Giantin (PRB-114C) was from Covance. All antobodies were diluted according to the manufacturer’s instructions. For immunofluorescences: fluorescent-conjugated secondary antibodies were from Life Technologies (AlexaFluor A21202, A21203, A21207), and diluted 1:1000. For western blot: HRP-conjugated secondary antibodies were from GE Healthcare (NA934V, NXA931), diluted 1:10000, and detected with ECL system (Amersham).

### Immunofluorescence

Cells plated on coverslips at low confluence or in wound-healing assay (see below) were fixed in 4% PFA-PBS or methanol for 10 min, permeabilized with 0.2% or 0.5% Triton X-100-PBS (for MEF or MDCK, respectively) and blocked with 3% BSA-PBS. Primary antibodies were diluted in blocking solution and incubated for 1 h at RT or ON at 4°C; subsequently, secondary antibodies or conjugated-antibodies were diluted in blocking solution and incubated for 1 h at RT; after, DAPI-PBS solution was addes for 10 min at RT; coverslips were mounted with Mowiol.

Digital images of representative fields were taken with Zeiss Axiophot or UltraView spinning disk confocal (PerkinElmer) microscope. Confocal Z-stacks were acquired and reconstructed with Volocity software.

### Migration assay

The experiment was performed as previously described [9]. Briefly, after filling the lower chamber of Boyden chambers with DMEM (with or without inhibitors), fibronectin-coated polycarbonate 8 μm-pore filters (Costar, Acton) were inserted, and 50000 cells in DMEM were added above, in the upper chamber; after 3 h (for fibroblasts) or O/N (MDCK/mIMCD) incubation cells on the upper surface of the filter were mechanically removed, while cells that pass to the bottom surface were fixed with ethanol, stained with Giemsa dye (Sigma, GS) and counted in 10 random fields per filter; fields have an approximate diameter of 0.5 mm. Within the experiment, each condition was plated in triplicate, and at least three independent experiments with the same conditions were performed.

### Wound-healing assay

The experiment was performed as previously described [9,18]. Briefly, cells were grown as high-density monolayers, scratched with a pipette tip, and after three washes to remove detached cells, allowed to migrate for the indicated time. For biochemical analysis: cells were grown on dishes at least O/N, multiple scratches in two perpendicular directions were performed, and after indicated time cells were lysed with 1% Triton X-100-lysis buffer. For immunofluorescences: cells were grown on coverslips in multiwells, 3–5 scratches were performed, and after indicated times cells were fixed in 4% PAF-PBS or methanol for immunofluorescence. For front-rear polarity quantification: cells were stained by immunofluorescence for Golgi and nucleus and front-rear polarized cells were counted: cells were considered polarized if they have re-positioned Golgi apparatus in front of the nucleus in a 120° angle towards the wound [18]. Each condition was plates in triplicate, and 3 groups of at least 100 cells were counted.

### Colorimetric adhesion assay

Cells were plated at a density of 60-70% and the day after resuspended and plated in 96 wells plates (70,000 MDCK cells/well; 100,000 MEF/mIMCD cells/well), previously coated with 1 μg/ml fibronectin in PBS for 1 h and blocked with 1% BSA-PBS 30 min; cells were forced to reach the bottom by spinning the plate (time 0). At different indicated times (10 min, 15 min, 30 min, 1 h, 3 h, 6 h), a wash with warm DMEM removed non-adherent cells, while remaining cells were fixed and colored with 0.5% crystal violet-20% methanol-H_2_O. Unbound dye was well washed and plate was left to dry hair. Equal amount of 1% SDS-H_2_O was added to the wells, and the concentration of the dye in the wells (proportional to the protein content, so to cell number) was read at the absorbance of 570 nm. Because PC-1 regulates cell size [33], we normalized each value with the absorbance value of unwashed cells 6 h after plating, representing 100% plated cells. Also, to each value blank/water absorbance value was substracted. Each condition was plated in triplicate.

### Nocodazole resistance assay

Cells were plated at low density and treated the day after with 2 μM nocodazole (45 min for MDCK cells, 30 min for MEF cells) to induce depolymerization of non-stable microtubules. After 3 minutes extraction in Triton X-100-PBS (0.2% for MDCK and mIMCD, 0.1% for MEF cells) at RT to remove monomeric tubulin, cells were fixed and immunofluorescence was performed for α-tubulin. Cells that remained with microtubule filaments were counted.

### Nocodazole washout assay

The experiment was performed as previously described [33]. Cells were plated at low confluence on fibronectin-coated coverslips and treated the day after with 10 μM nocodazole for 4 h to induce complete depolymerization of microtubules. Nocodazole was washed and fresh medium (with or without inhibitors) was added (time 0). At different indicated time (15 min, 30 min, 1 h, 2 h) cells were fixed with 4% PFA-PBS, and immunofluorescence for actin, tubulin and paxillin was performed. Cells containing focal adhesion (with at least 10 visible focal adhesions) were counted; at least 100 cells for every condition were counted.

### Fluorescence Recovery after Photobleaching (FRAP) of paxillin-GFP

Fluorescence Recovery After Photobleaching (FRAP) was performed as described in [24] on an UltraVIEW VoX spinning disc confocal system (PerkinElmer), equipped with an EclipseTi inverted microscope (Nikon) provided with a Nikon Perfect Focus System, an integrated FRAP PhotoKinesis unit (PerkinElmer), a C9100-50 emCCD camera (Hamamatsu) and driven by Volocity software (Improvision, Perkin Elmer).

Cells were placed in an environmental microscope incubator (OKOLab) set to 37°C and 5% CO_2_ perfusion. All images were acquired through a 60× oil immersion objective (Nikon Plan Apo VC, NA 1.4). Cells were transiently transfected, 48 hours before FRAP, with GFP-paxillin and plated on glass 24 hours before imaging. From 2 to 5 bleach regions, corresponding to selected focal adhesions, with a size of 4 × 2 μm were positioned on cells. Photobleaching was performed using fifty iterations with the 50 mW solid state 488 nm laser set to the maximum power. We calculated the efficiency of the bleaching process as the difference between the mean fluorescence intensity in the FA area before the bleach and at the first post-bleach time point, normalized with respect to the first. The percentage of bleaching efficiency calculated on a sample of 20 FAs was 86,3 ± 8,8 (mean value ± SD). To determine the recovery kinetics of peripheral adhesions, post-bleaching images were recorded for 350 seconds: the first 60 seconds with a speed of 1 frame/sec and then of 0.2 frame/sec. Quantitative analyses were performed with ImageJ software: the mean intensity values over time were measured, background subtracted and corrected for acquisition photobleaching. A single exponential function was used to fit the recovery curves of focal adhesions.

### Statistical analysis

Differences between averages were established with Student’s T-test or one way ANOVA analysis of variance, as indicated in the figure legends; Bonferroni’s post-test was carried out for multiple comparisons.

### Ethical approvement

The cell lines used in this paper were either commonly employed cell lines or mouse embryonic fibroblasts described in previous papers [32]. No reagents that require an ethical statement were employed.

### Availability of supporting data

The data sets supporting the results on this article are included within the article (and its additional files).

## Additional files

Additional file 1: Figure S1. Pkd1 gene silencing in murine IMCD cells. **Figure S2** Inhibition of PI-3 kinase decreases PC-1-dependent cell migration but not PC-1-dependent front-rear polarity. **Figure S3** PC-1 overexpression promotes focal adhesions formation during the first steps of adhesion. **Figure S4.** Pkd1 knock-out fibroblasts display defective focal adhesion formation during migration. **Figure S5.** PC-1 overexpression promotes focal adhesion formation and orientation at the leading edge of migrating cells. **Figure S6.** Polycystin-1 overexpression promotes focal adhesion disassembly, while knock-out of Pkd1 gene decrease their dynamics. Additional file 2: Movie S1. FRAP analysis of GFP-paxillin turnover in control MDCK^*Zeo*^ cell and in *PKD1*-overexpressing MDCK^*PKD1Zeo*^ cell. Additional file 3: Movie S2. FRAP analysis of GFP-paxillin turnover in one selected FA from control MDCK^*Zeo*^ and *PKD1*-overexpressing MDCK^PKD1Zeo^ cells. Additional file 4: Movie S3. FRAP analysis of GFP-paxillin turnover in control *Pkd1* ^+/+^ and in *Pkd1* ^−/−^ fibroblasts. Additional file 5: Movie S4. FRAP analysis of GFP-paxillin turnover in one selected FA from *Pkd1* ^+/+^ and *Pkd1* ^−/−^ fibroblasts.
