# Supplementary material for: Regulation of the microtubular cytoskeleton by Polycystin-1 favors focal adhesions turnover to modulate cell adhesion and migration
Source: BMC Cell Biol. 2015 May 7;16:15. doi: 10.1186/s12860-015-0059-3 (PMC4437554; doi:10.1186/s12860-015-0059-3)
Supplement: Additional file 1: Figure S1. — Pkd1 gene silencing in murine IMCD cells. Figure S2 Inhibition of PI-3 kinase decreases PC-1-dependent cell migration but not PC-1-dependent front-rear polarity. Figure S3 PC-1 overexpression promotes focal adhesions formation during the first steps of adhesion. Figure S4. Pkd1 knock-out fibroblasts display defective focal adhesion formation during migration. Figure S5. PC-1 overexpression promotes focal adhesion formation and orientation at the leading edge of migrating cells. Figure S6. Polycystin-1 overexpression promotes focal adhesion disassembly, while knock-out of Pkd1 gene decrease their dynamics. [file 12860_2015_59_MOESM1_ESM.pdf]

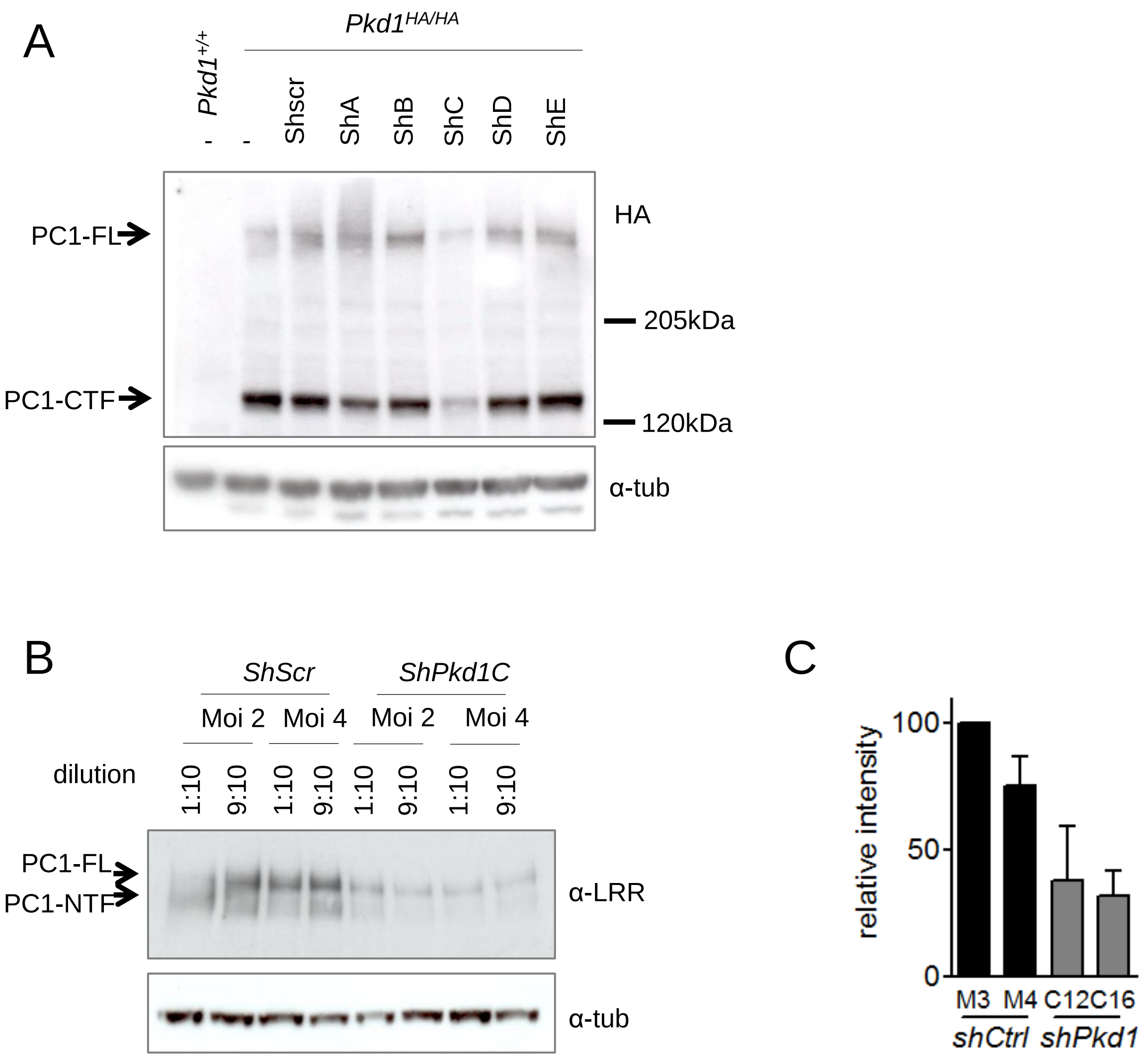

Figure S1. Pkd1 gene silencing in murine IMCD cells

(**A**) Screening of Pkd1 shRNAs was performed transducing Pkd1HA/HA murine fibroblasts (REF 20) with viral vectors expressing shRNA encoding scrambled (shScr) sequences or 6 different murine Pkd1-targeting sequences(sh A-E). Cell lysates were tested for PC-1 protein expression by western blot analysis with HA antibody; α-tubulin is used as loading control. This screening identified shPkd1C sequence as the most efficient on murine Pkd1. (**B**) Silencing of Pkd1 on mIMCD cells was performed by transducing cells with shPkd1C, as well as with control shScr, subsequently splitted using two different dilutions (1:10, 9:10) and selected for puromycin resistance. After 5 days of selection, cell lysates were analysed by western blot analysis using PC-1 (LRR) antibody; α-tubulin is used as loading control. Resistant cells treated with MOI 4 and diluted 9:10 were chosen for subcloning.(**C**) PC-1 protein expression levels were analysed in the lysates of the four chosen clones (M3, M4, C12, C16; Figure 1), and quantified by densitometry. Histograms represent the average of densitometry values and standard error of the mean (SEM).

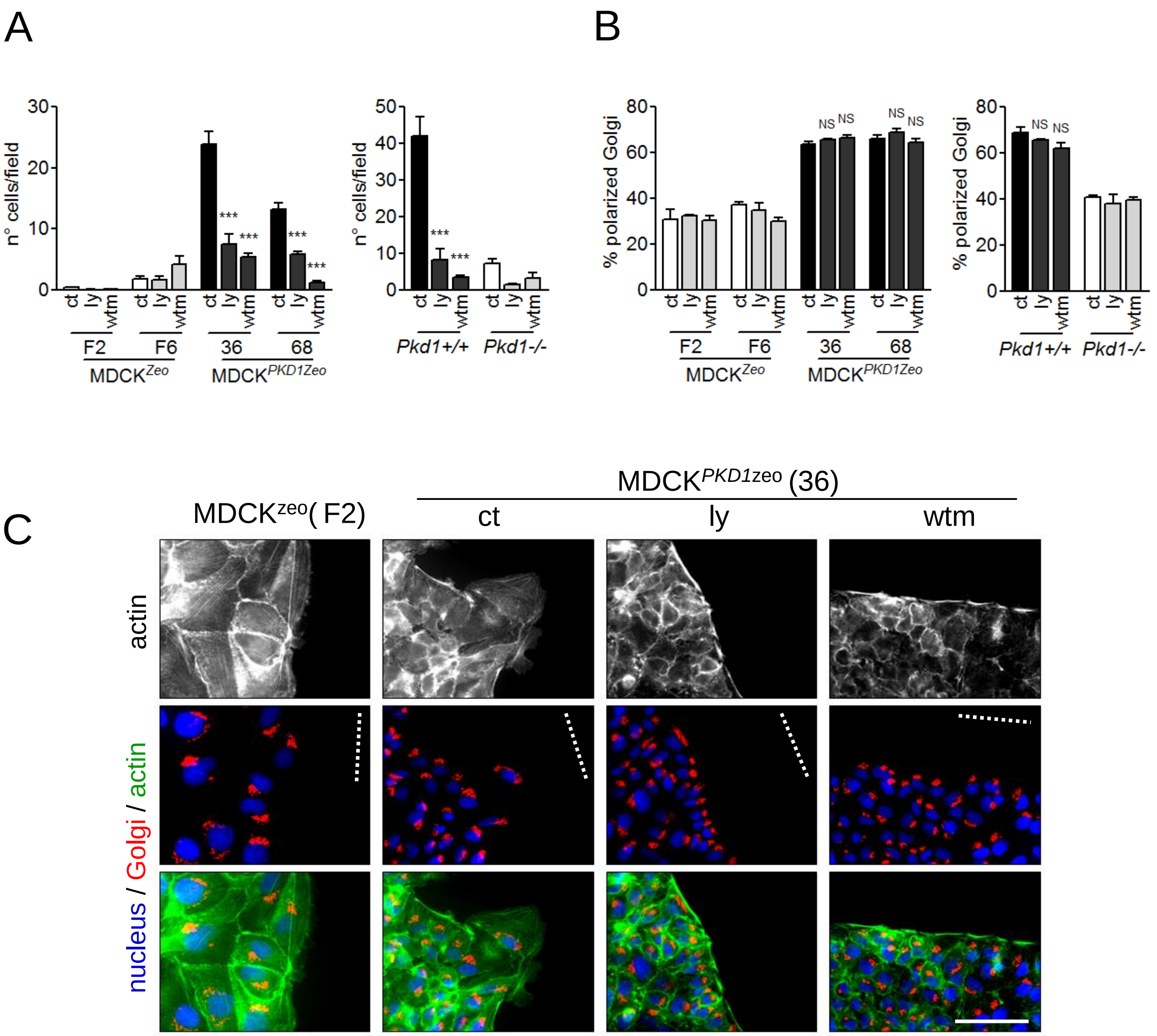

Figure S2. Inhibition of PI-3 kinase decreases PC-1-dependent cell migration but not PC-1-dependent front-rear polarity.

(**A**) Boyden chambers assays on MDCK cells (left panel) and fibroblasts (right panel) was carried out in the presence of LY294002 (ly) or Wortmannin (wtm), revealing the role of PI-3 kinase in cell migration. Statistical analysis: ANOVA; ***$p<0.001$, referred to the relative control (ct) bar. (**B**) Quantification of Golgi repositioning revealed no effect of LY294002 or Wortmannin on front-rear polarity in MDCK^{PKD1Zeo} cells or wt fibroblasts. Statistical analysis: ANOVA; NS non statistically significant ($p>0.05$), referred to the relative control (ct) bar. (**C**) MDCK^{Zeo} (clone F2) and MDCK^{PKD1Zeo} (clone 36) were subject to wound healing, allowed to migrate for three hours in the presence or absence of LY294002 or Wortmannin; staining with phalloidin (actin), anti-giantin (Golgi) and DAPI (nucleus) clearly shows effects of the inhibitors on actin. Bar: 50µm.

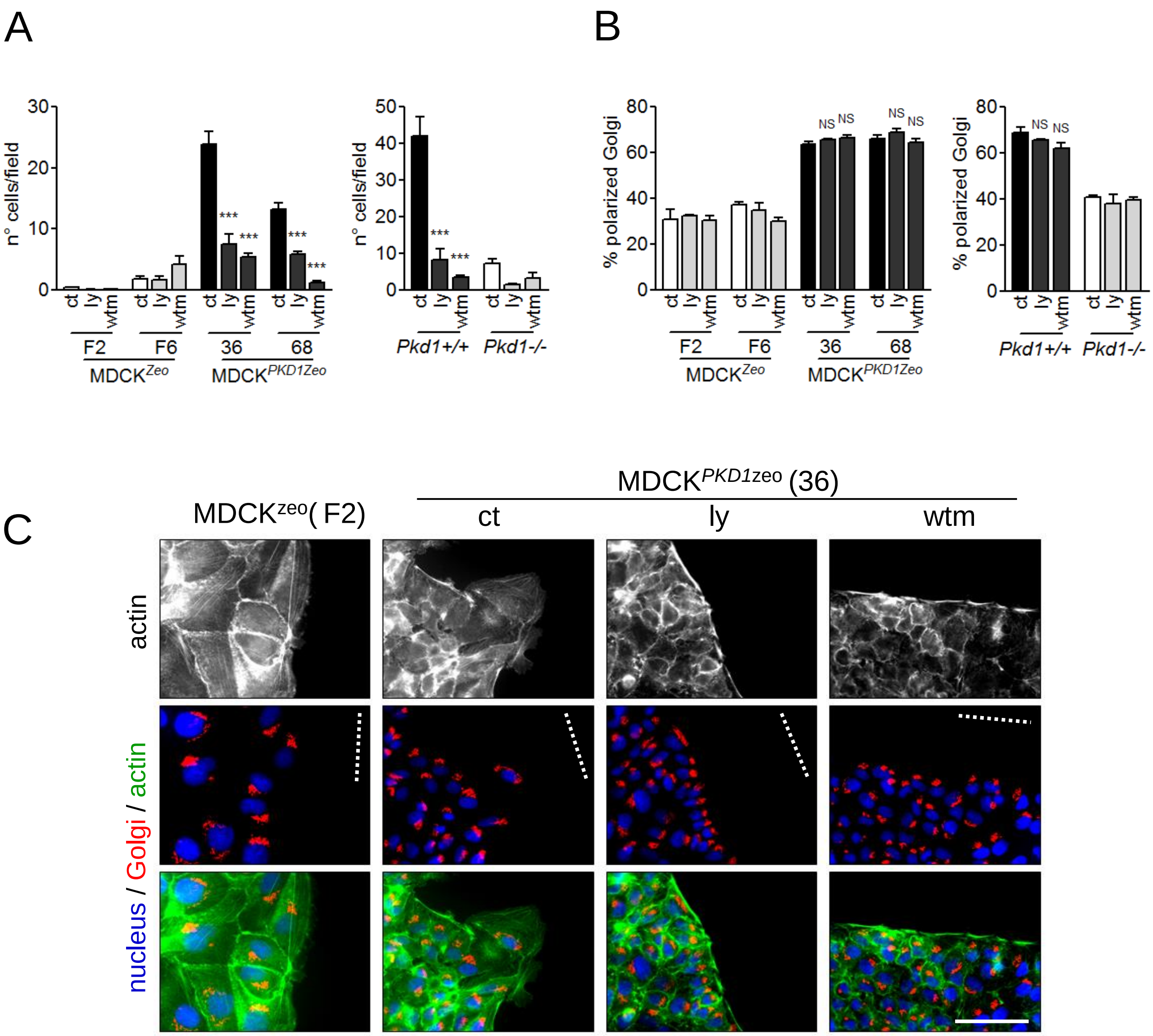

Figure S2. Inhibition of PI-3 kinase decreases PC-1-dependent cell migration but not PC-1-dependent front-rear polarity.

(**A**) Boyden chambers assays on MDCK cells (left panel) and fibroblasts (right panel) was carried out in the presence of LY294002 (ly) or Wortmannin (wtm), revealing the role of PI-3 kinase in cell migration. Statistical analysis: ANOVA; ***p<0.001, referred to the relative control (ct) bar. (**B**) Quantification of Golgi repositioning revealed no effect of LY294002 or Wortmannin on front-rear polarity in MDCK^{PKD1Zeo} cells or wt fibroblasts. Statistical analysis: ANOVA; NS non statistically significant (p>0.05), referred to the relative control (ct) bar. (**C**) MDCK^{Zeo} (clone F2) and MDCK^{PKD1Zeo} (clone 36) were subject to wound healing, allowed to migrate for three hours in the presence or absence of LY294002 or Wortmannin; staining with phalloidin (actin), anti-giantin (Golgi) and DAPI (nucleus) clearly shows effects of the inhibitors on actin. Bar: 50μm.

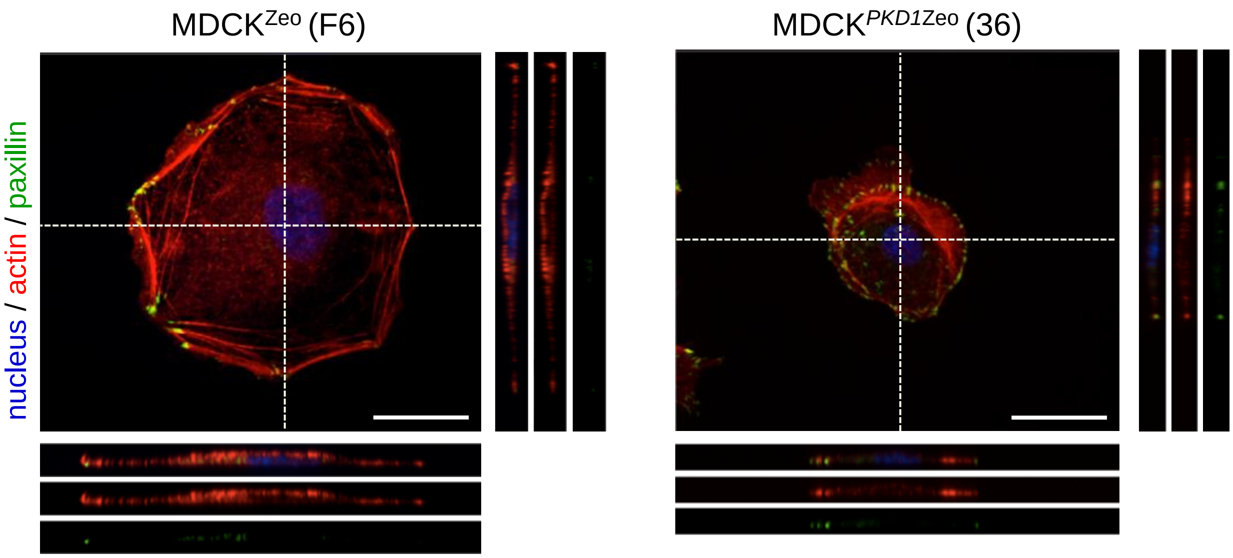

Figure S3. PC-1 overexpression promotes focal adhesions formation during the first steps of adhesion.

Confocal images of immunofluorescence on MDCK^{Zeo} (clone F6) and MDCK^{PKD1Zeo} (clone 36), 1 hour after plating on fibronectin. Cells were stained for actin (Phalloidin-TRITC, red), paxillin (green) and nucleus (DAPI, blue). Big images represent one confocal Z-section of the cell, on the right and below each image are projections along x and y axis, reconstructed with Volocity software. In all cell lines, paxillin staining is found on the ventral side and localises in clusters (focal adhesions). Bar: 25 µm.

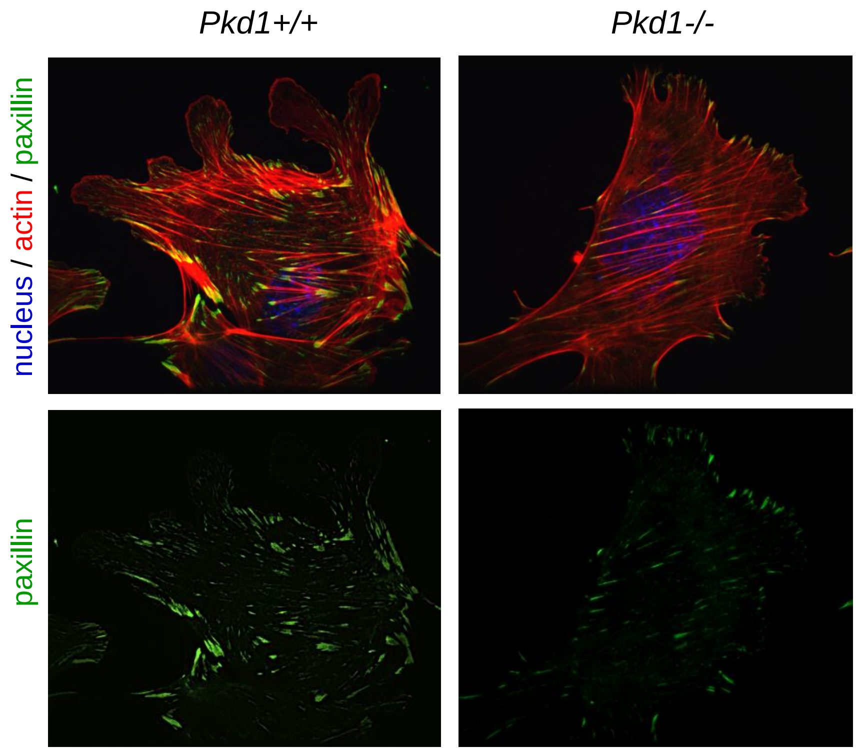

Figure S4. Pkd1 knock-out fibroblasts display defective focal adhesion formation during migration.

Immunofluorescence of *Pkd1*$^{+/+}$ and *Pkd1*$^{-/-}$ fibroblasts migrating on fibronectin. Cells were stained for actin (Phalloidin-TRITC, red), paxillin (green) and nucleus (DAPI, blue).

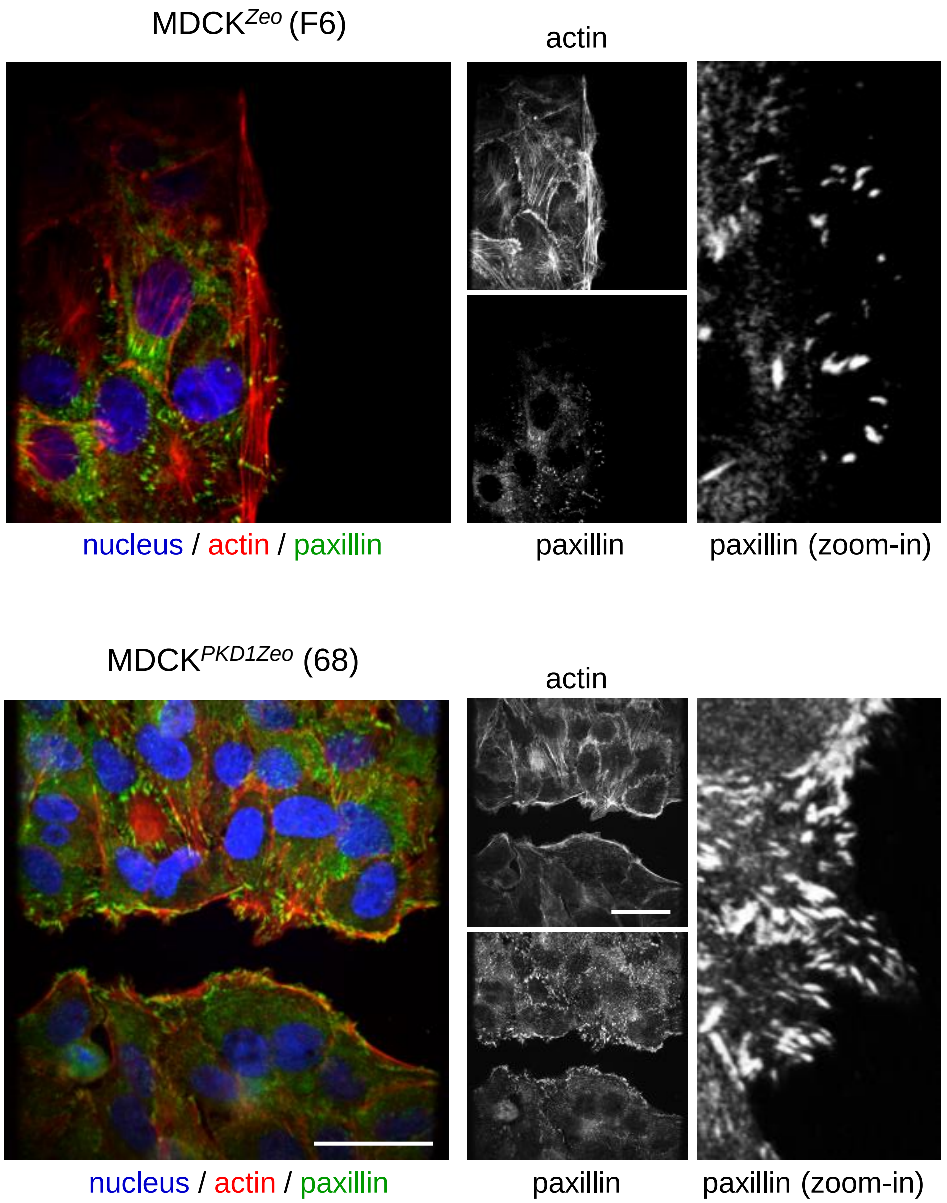

Figure S5. PC-1 overexpression promotes focal adhesion formation and orientation at the leading edge of migrating cells.
Representative confocal images of MDCK^{Zeo} (clone F6), and MDCK^{PKD1Zeo} (clone 68) cells, allowed to migrate in a 3 hours wound-healing assay on fibronectin and subsequently stained for actin (Phalloidin-TRITC), paxillin (green) and nucleus (DAPI, blue). Upper panels show merged images, lower panels show each single channel and zoom-in images of paxillin magnified 3 times. Bar: 25µm.

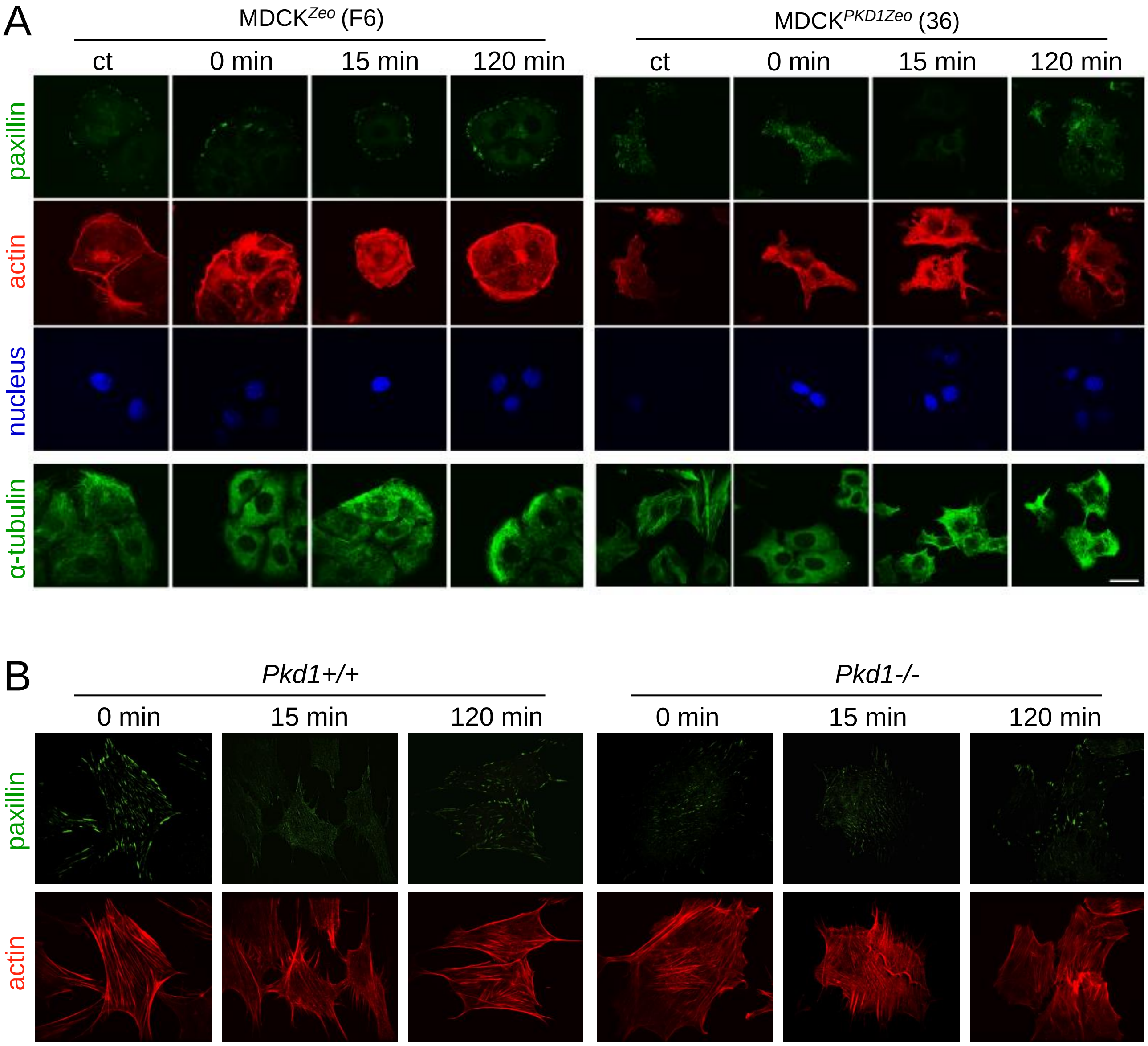

Figure S6. Polycystin-1 overexpression promotes focal adhesion disassembly, while knock-out of Pkd1 gene decrease their dynamics.

(**A**) Immunofluorescence images of MDCK^{Zeo} (clone F6) and MDCK^{PKD1Zeo} (clone 36) cells during nocodazole washout assay. Cells were stained for actin (phalloidin-TRITC), paxillin (green) and nucleus (DAPI, blue). Control microtubule staining is showed (green). Nocodazole treatment (4h, 10µM) depolymerises completely microtubules and cells maintain their focal adhesion; 15 minutes after nocodazole washout, microtubules are repolimerised in all clones. In MDCK^{PKD1Zeo} cells, focal adhesions are lost in the majority of cells and restored completely 120 minutes after washout. As a control: cells not treated with nocodazole are shown (ct). Bar: 25 µm. (**B**) Immunofluorescence images *Pkd1*$^{+/+}$ and *Pkd1*$^{-/-}$ cells during the nocodazole washout assay (as in A). 15 minutes after nocodazole washout, a higher percentage of focal adhesions is lost in *Pkd1*$^{+/+}$ cells, compared to *Pkd1*$^{-/-}$ cells; focal adhesions are restored completely 120 minutes after washout. Bar: 25 µm.

Movie S1. FRAP analysis of GFP-paxillin turnover in control MDCK^{Zeo} cell and in *PKD1*-overexpressing MDCK^{PKD1Zeo} cell.

Time-lapse movies from FRAP analysis of GFP-paxillin-transfected MDCK^{Zeo} (clone F6; on the left) and MDCK^{PKD1Zeo} (clone 36; on the right) cells. Time is in seconds, relative to the time of photobleaching T=0. Bar: 10 µm. Playback is 5 frames/sec.

Movie S2. FRAP analysis of GFP-paxillin turnover in one selected FA from control MDCK^{Zeo} and *PKD1*-overexpressing MDCK$^{\mathrm{PKD1Zeo}}$ cells.

Zoom in of bleached focal adhesions from the MDCK^{Zeo} (clone F6; on the left) and MDCK^{PKD1Zeo} (clone 36; on the right) cells shown in Movie S1. Time is in seconds, relative to the time of photobleaching T=0. Bar: 2 µm. Playback is 5 frames/sec. Significant images are shown in Figure 5C.

Movie S3. FRAP analysis of GFP-paxillin turnover in control *Pkd1*$^{+/+}$ and in *Pkd1*$^{-/-}$ fibroblasts.

Time-lapse movies from FRAP analysis of GFP-paxillin-transfected *Pkd1*$^{+/+}$ (on the left) and *Pkd1*$^{-/-}$ (on the right) cells. Time is in seconds, relative to the time of photobleaching T=0. Bar: 10 µm. Playback is 5 frames/sec.

Movie S4. FRAP analysis of GFP-paxillin turnover in one selected FA from *Pkd1*$^{+/+}$ and *Pkd1*$^{-/-}$ fibroblasts.

Zoom in of bleached focal adhesions from the *Pkd1*$^{+/+}$ (on the left) or *Pkd1*$^{-/-}$ (on the right) cells shown in Movie S3. Time is in seconds, relative to the time of photobleaching T=0. Bar: 2 µm. Playback is 5 frames/sec. Significant images are shown in Figure 5C.
